# Awareness and knowledge of canine rabies: A state-wide cross-sectional study in Nigeria

**DOI:** 10.1371/journal.pone.0247523

**Published:** 2021-03-03

**Authors:** Ahmad I. Al-Mustapha, Abubakar A. Tijani, Folashade O. Bamidele, Oyewo Muftau, Ahmed Ibrahim, Ibrahim Abdulrahim, Muhammad Shuaib Osu, Grace Kia, Nguku Patrick, Waziri N. Endie

**Affiliations:** 1 Department of Veterinary Services, Kwara State Ministry of Agriculture and Rural Development, Ilorin, Nigeria; 2 Department of Veterinary Public Health and Preventive Medicine, Faculty of Veterinary Medicine, University of Ibadan, Ibadan, Oyo State, Nigeria; 3 Nigerian Field Epidemiology and Laboratory Training Program, Asokoro, Abuja, Nigeria; 4 Federal Department of Veterinary and Pest Control Services, Federal Ministry of Agriculture and Rural Development, Abuja, Nigeria; 5 Department of Veterinary Public Health and Preventive Medicine, Faculty of Veterinary Medicine, Ahmadu Bello University, Zaria, Kaduna State, Nigeria; 6 War Against Rabies Foundation, Abuja, Nigeria; 7 African Field Epidemiology Network, Abuja, Nigeria; University of Lincoln, UNITED KINGDOM

## Abstract

Rabies is a highly fatal disease that is endemic in Nigeria. The poor community awareness and knowledge of canine rabies have thwarted the realization of zero deaths from dog mediated human rabies. This study aimed to assess the awareness and knowledge of canine rabies in Kwara state. A total of 1,460 questionnaires were administered to respondents in the three senatorial zones of the state using open data kit (ODK) on mobile phones between September 2019 to January 2020. The rabies awareness rate was 38.1%. The mean knowledge score was 3.78 ± 2.15. Only 10.6% (n = 59/557) of the respondents had satisfactory knowledge of canine rabies. Respondents had poor knowledge of the mode of transmission, symptoms, prevention, and the control measures needed to eliminate canine rabies. Only 20.1% of respondents owned at least a dog. Dog owners were 3.85× (95% CI: 2.89, 5.13; p < 0.01) more likely to be aware of canine rabies and were 1.78× (95% CI: 1.22–2.60; p = 0.003) more likely to have satisfactory knowledge about canine rabies than non-dog owners. Respondents with tertiary education were at least 6.81× (95% CI: 4.24, 10.92; p < 0.01) more likely to be aware of rabies than respondents with no formal education. The findings of this study showed very low awareness and knowledge of canine rabies among residents of Kwara state. Mass sensitization of the populace on the dangers of rabies should be intensified. Such interventions should be targeted at the general public and dog owners.

## Introduction

Rabies is a viral disease of mammals that is endemic in Africa and Asia [1]. It is a neglected tropical disease that is under-funded, under-diagnosed, and under-reported in Nigeria [2, 3]. Rabies is responsible for approximately 59,000 deaths globally and costs over USD 8.6 billion annually [4, 5]. Nigeria has an estimated ten thousand dog-bite incidents annually [6] and some 1,640 estimated human rabies cases each year [5, 7]. To reduce the incidence of dog-bites and prevent dog-mediated human rabies, it is essential to assess the awareness level and further educate the general public. This has proved effective in changing the risk perception of the populace and resulted in attitudinal changes that were necessary to reduce the burden of rabies [8, 9].

A myriad of challenges such as poor awareness and knowledge of rabies, lack of political will, shortage of vaccines, and other vaccine-related factors has led to the endemicity of rabies and significant under-reporting of rabies in Nigeria [10]. Moreover, the presence of free-roaming dogs has complicated rabies elimination in Africa and Asia [11–13]. Globally, eradication of canine rabies combined with post-exposure prophylaxis is widely considered as the most cost-effective means of eradicating human rabies [14, 15].

The poor rabies awareness rates amongst the general public including policymakers have undermined the public health importance of rabies [16, 17]. In Kwara State and Nigeria at large, there is no national or state-based rabies control program in force. Hence, the proposed Kwara Rabies Rapid Alert System (KRRAS) was designed to serve as a pioneer one-health framework for rabies elimination in the state. KRRAS will focus on the following thematic areas: public enlightenment, mass vaccination campaigns, enhanced surveillance of dog-bite incidents in the state, and reporting of dog-bite incidents to the district health information system (DHIS II) of the state.

Responsible dog ownership (good health, welfare, feeding, and vaccination) and stray dog population control methods are of prime importance in reducing dog-mediated human rabies [14]. Also, regulations such as compulsory registration of dogs, mandatory annual vaccinations will help in eliminating the menace of rabies [15].

Although several dog ecology studies had estimated dog population, demography, and evaluated rabies control in Kwara State [18–20], there is still a paucity of data on the knowledge and practices of the general populace on canine rabies. Hence, this study was conducted as a baseline investigation of public awareness about canine rabies in Kwara state.

## Materials and methods

### Ethics statement

This study was conducted in accordance with the requirements of the Nagoya protocol. Ethical clearance was obtained from the ethical review boards of the Kwara State Ministry of Health (reference number MOH/KS/EU/777/31), and the Ministry of Education and Human Capital Development, Ilorin—Nigeria (reference number: DE/PRIM/96/VOL.1/130). Written informed consent was sought from each respondent (occasionally from heads of a household) and participants could decline participation and opt-out at any time.

### Study area

Kwara State, located in the southern guinea savannah zone of Nigeria between latitude 9.394871°N & 8.655247°N, and longitude 3.101850°E & 4.387415°E. The state has a human population of 3,599,800 [21]. The state has three agro-ecological and geographical zones (Northern, Central, and Southern) with vast land and with varying climatic conditions.

### Period and course of the survey

The cross-sectional survey was carried out using a structured pre-validated questionnaire. The questionnaire was composed of four sections: a) Owner demographics b) Awareness of canine rabies c) Knowledge of rabies d) Practices associated with dog management (S1 File). Section B contained a single question (Are you aware of rabies?). Hence, we applied skip logic, so that questions on in depth knowledge of rabies were skipped for respondents who were not aware of rabies. The questionnaire was validated by two independent examiners to verify its content and face validity. It was then pre-tested on ten respondents before its deployment for field use to assess any technical hitches.

The survey was conducted from September 2019 to January 2020 in the three senatorial zones of Kwara state. A multi-stage sampling (S1 Fig) of the respondents was carried out from all the communities in the state. Furthermore, a systematic random sampling of households was conducted to select respondents in each street. In rural communities where there are no organized streets, the polio micro-plan house markings were used as a guide [22, 23]. A sampling interval of 5 houses was used for this study. A team of volunteers were trained, mobilized, and assigned separate predetermined routes. The survey was conducted on each chosen street. Starting from the first houses on the right side, every 5th house was selected and an adult member was interviewed using the Open Data Kit (ODK).

The questionnaire was administered as a one-on-one interview. Hence, the intra-cluster correlation was not considered during the estimation of the effective sample size, which was computed using Epi-Info V.7.0 (CDC, Atlanta, USA). At a 95% confidence interval (95% CI), we hypothesized that 50% of the respondents will have prior knowledge of rabies in each senatorial zone (384 respondents per zone, 1152 for the state). Furthermore, we added a 25% non-response rate to the total required participants for the state (n = 288). Therefore, the minimum number of households included in this survey was 1,440.

### Data analysis

The data were analyzed using Minitab version 19.1.1 (Pennsylvania, USA). Descriptive statistics of demographic data were summarized as frequencies and proportions. Each question was allotted a point when answered correctly. Only respondents who were aware of rabies, were interviewed on their knowledge of canine rabies. The canine rabies knowledge score was computed to determine the knowledge of canine rabies for each respondent. The general knowledge of canine rabies was graded based on 50% of the maximum obtainable score. Hence, respondents who were aware of rabies but had a score greater than 50% of the maximum obtainable score were considered to have a satisfactory knowledge of canine rabies and vice versa (Table 1) [24]. To determine the predictors of high awareness rates and knowledge levels of canine rabies, variables were subjected to a logistic regression analysis at p<0.05.

**Table 1:** Rabies awareness and knowledge among residents of Kwara state (n = 1460).

Variables	Frequency (%)
Heard of rabies (locally called digbolugi, hawkan kare, gben bande)?	
No	903 (61.9)
Yes	557 (38.2)
Cause of rabies	
Bacteria	95 (17.1)
Fungi	21 (3.8)
I don’t know	285 (51.2)
Protozoan	31 (5.6)
Virus	125 (22.4)
Symptoms of Rabies	
Behavioural Changes	438 (79)
Dropped jaw	30 (5)
Fever	100 (18)
Hydrophobia	10 (1.8)
I don’t know	88 (15.8)
Inability to swallow	42 (7.5)
Paralysis	19 (3.4)
Pica	51 (9.2)
Seizures	12 (2.2)
Mode of transmission	
Blood	84 (15)
Contact	16 (2.8)
Dog bites	476 (85.4)
Penetration of open wound with dog saliva	56 (10)
I don’t know	60 (10.7)
Preventive Measures	
Antibiotics	58 (10.4)
Human vaccinations	45 (8)
Killing stray dogs	216 (38.7)
Mass dog vaccinations	253 (45.4)
I don’t know	96 (17.2)
Control Measures	
Killing of stray-dogs	187 (33.5)
Mass dog vaccinations	238 (42.7)
Public awareness campaigns	151 (27.1)
Spaying	9 (1.6)
I don’t know	85 (15.2)

## Results

### Demography of respondents

The total number of respondents was 1,460 (S2 Fig). Of these, 72% had either secondary or tertiary education which made administering the survey in English feasible. However, the questionnaire was administered in the local language of the community when needed (Table 2).

**Table 2:** Demographic data for respondents in the three senatorial zones of Kwara state (n = 1460).

Variables	Frequency (%)
Senatorial zone	
Kwara North	562 (38.5)
Kwara Central	534 (36.5)
Kwara South	364 (25)
Gender	
Male	1072 (73.5)
Female	382 (26.1)
Prefer not to say	6 (0.4)
Level of Education	
No western education	187 (13.5)
Primary education	165 (11.9)
Secondary	581 (41.9)
Tertiary	424 (30.6)
Others	30 (2.1)
Prefer not to say	73 (5)

### Rabies awareness and knowledge among residents of Kwara state

Only 38% (n = 557/1460) of the respondents have heard of rabies. The mean knowledge score was 3.78 ± 2.15. With a maximum obtainable score of 15 points, study participants obtained knowledge scores ranging from 0 to 13. Using the 50% of the maximum obtainable score as the cut-off, only 10.6%, n = 59/557) of the respondents had a satisfactory (≥ 7.5) knowledge of canine rabies. Only 22.4% (n = 125/557) of respondents knew that rabies was caused by a virus. Similarly, 85.4% (n = 476/557) knew that human rabies is mostly caused by dog bites. However, only 10% of respondents (n = 56/557) knew that rabies can be transmitted through the saliva of dogs when it comes in contact with open wounds. The majority of the respondents (92.2%; n = 514/557) knew at least one preventive measure against rabies. Of these respondents, 45.4% (n = 253/557) of respondents knew that mass dog vaccination was an effective preventive measure against the transmission of dog -mediated human rabies. However, 38.7% (n = 216/557) of respondents thought the killing of stray dogs is an effective means of preventing human rabies. Only 13% (n = 73/557) knew at least two correct preventive measures. All respondents knew at least a control measure for rabies in dogs, while 16.3% (n = 91/557) knew more than one control measure.

### Practices associated with dog ownership

The management of dog-bite incidents of most respondents were positive. Most of the respondents (65.7%; n = 960/1460) took dog-bite wounds to a health facility. However, 20.9% (n = 305/1460), 1.8% (n = 27/1460) and 1.7% (n = 25/1460) of respondents still believed in traditional concoctions, spiritual interference or did nothing to dog-bite incidents respectively. About 30.5% (n = 446/1460) of the respondents treat dog-bite wounds themselves without going to any health facility. This treatment includes self-prescription of antibiotics, analgesics, ointments, or the use of hot water to clean wound surfaces, and bites were managed as open wounds. Only 20% (n = 293/1460) of households had at least one dog during the survey. Of these, 27% (n = 80/293) of owned dogs have always been confined within cages or the perimeter fence. There were 17% (n = 50/293) of dogs partially confined (confined during the day and released at night) whereas 52% (n = 152/293) of dogs were never confined. Most dogs (70%, n = 200/286) were cared for by everyone in the household and the majority (81%, n = 238/293) were fed family leftovers and a few dogs (7%, n = 20/293) were fed commercial dog feed. Many dogs (51%, n = 154/293) were obtained as gifts from friends and family. Similarly, several dogs were depopulated by giving them away (55%, n = 36/65). Some dog owners 23% (n = 15/65) sold their puppies. Sixty four percent of the respondents were satisfied with the quality of veterinary services they received (S1 Table).

### Analysis of variables affecting awareness and knowledge of rabies in Kwara state

Dog ownership, age of the respondents, and their level of education had a significant impact on the respondents’ awareness of rabies. Dog owners were 3.85 × (95% CI: 2.89, 5.13; p < 0.001) more likely to be aware of rabies. In addition, they were more likely (OR: 1.78; 95% CI: 1.22–2.60; p = 0.003) to have satisfactory knowledge of canine rabies than respondents with no dogs. Respondents with tertiary education were 6.81 × (95% CI: 4.24, 10.92; p < 0.001) more likely to be aware of rabies than respondents with no formal education (Table 3). However, religion, gender, and the number of persons per household did not affect rabies awareness or the knowledge of rabies (Table 3).

**Table 3:** Univariate and multivariate logistic regression analysis of demographic variables affecting awareness and knowledge of canine rabies in Kwara state.

Outcome variable	Variable	Referent		OR (95% CI)	*p-value*	OR (95% CI)	*p-value*
		Univariate analysis				Multivariate analysis	
Rabies Awareness	Age	<18 years	18–28	0.98 (0.52, 1.87)	0.001	1.10 (0.54, 2.16)	<0.001
			29–38	0.67 (0.35, 1.28)		0.85 (0.43, 1.71)	
			39–48	0.89 (0.46, 1.75)		1.14 (0.56, 2.33)	
			49–58	1.20 (0.58, 2.50)		2.35 (1.05, 5.26)	
			>59	2.45 (1.10, 5.44)		4.78 (1.97, 11.57)	
	Level of Education	No formal education	Primary	2.94 (1.84, 4.69)	0.001	3.52 (2.10, 5.88)	< 0.001
			Secondary	2.20 (1.49, 3.25)		3.34 (2.10, 5.29)	
			Tertiary	3.83 (2.57, 5.72)		6.81 (4.24, 10.92)	
			Others	0.95 (0.36, 2.48)		1.83 (0.66, 5.04)	
	Dog ownership	No	Yes	3.59 (2.75, 4.69)	0.001	3.85 (2.89, 5.13)	< 0.001
	Gender	Female	Male	0.83 (0.65, 1.05)	0.121	-	
	Humans/households	1–4	5–7	1.04 (0.83, 1.30)	0.915	-	
			>7	1.08 (0.75, 1.54)			
Rabies Knowledge	Age	<18 years	18–28	0.70 (0.15, 3.38)	0.510	-	
			29–38	0.81 (0.17, 3.89)			
			39–48	0.86 (0.18, 4.28)			
			49–58	0.30 (0.04, 2.36)			
			>59	0.32 (0.04, 2.56)			
	Level of Education	No formal education	Primary	1.10 (0.63, 1.91)	0.829	-	
			Secondary	0.84 (0.54, 1.31)			
			Tertiary	0.87 (0.51, 1.50)			
			Others	0.97 (0.06, 15.81)			
	Dog ownership	No	Yes	1.78 (1.23, 2.60)	0.003	-	-
	Gender	Female	Male	0.98 (0.66, 1.45)	0.921	-	
	Humans/households	1–4	5–7	1.05 (0.59, 1.10)	0.393	-	
			>7	0.43 (0.09, 1.89)			

%—Percentage; α = 0.25; OR–Odds ratio; 95% CI– 95% confidence interval.

## Discussion

For any successful disease control program, it is essential to generate a context-specific evidence base that will highlight the key gaps that will be addressed during the intervention program. For effective rabies control in Kwara State, the awareness rate and the knowledge level of rabies must be improved. This low awareness rate of canine rabies is worrying. This low awareness rate among the populace might be due to the lack of prioritization of rabies by the government and the lack of a state-wide or a national rabies control program in Nigeria. The rabies awareness rate in this study was very low compared to the 82% awareness rate reported in Abuja, Federal Capital Territory [25] as well as the 76.5% and 71.5% reported from Nasarawa state [26] and Taraba state [27] respectively. It is also lower than the awareness rates reported from other parts of the world. For instance, studies from Bangladesh [28]; Ethiopia [29]; Sri Lanka [30] showed 73.0%, 75.2%, and 78.7% rabies awareness levels respectively. A significantly higher rabies awareness rate of 99% had been reported by other studies from Ethiopia [31] and Guatemala [32]. This might be due to the establishment of effective national rabies control programs in these countries. Globally, sensitizing the populace has increased awareness levels which have resulted in significant improvements in rabies control programs [30, 33].

A mean knowledge score of canine rabies was also very poor. Of the respondents, only some knew that rabies was caused by a virus. This is lower than the 47.5% reported by Christopher et al. [26]. On the contrary, most respondents knew at least one symptom of rabies (locally called digbolugi, hawkan kare, gben bande). Behavioral changes exhibited by rabid dogs were the most noticed by the populace, while a few others knew that rabies (dumb form) could be characterized by paralysis. This implies that some rabies cases might have been missed due to ignorance of the dumb form of rabies. In Nigeria, dog bite is the primary mode of rabies transmission (99%). However, because the virus is being carried in the saliva, it is possible that rabies can be transmitted through the saliva of dogs when it comes in contact with open wounds. This is an occupational hazard for veterinarians and un-vaccinated dog owners that might have open wounds [34]. Our study shows that only very few respondents (10%) were aware that rabies can be transmitted through the saliva of dogs when it comes in contact with open wounds.

Many of the respondents thought that killing stray dogs is an effective preventive measure to reduce the chances of dog bite incidents and the potential risk of human rabies. Similarly, some of the respondents thought that by killing stray dogs, we can control rabies in dogs. This is a wrong perception that needs to be corrected. Globally, reproduction control, movement restriction, and habitat control are the three practical methods of dog population management [35, 36]. However, the high reproductive rate of dogs had surpassed the highest recorded stray dog removal program [35]. There was no evidence from any rabies control program in endemic countries that stray dog removal alone had a significant impact on the spread of rabies [37, 38]. However, the WHO reported indirect benefits from selective elimination of unvaccinated dogs around human gatherings such as abattoirs and markets [35].

Most of the respondents knew at least one preventive or control measure against canine rabies. They also knew that mass dog vaccination was an effective preventive measure against the transmission of dog-mediated human rabies. This is vital to our rabies control program as it improves the presentation of dogs during mass vaccination campaigns. Most of the dog owners (78%, n = 228/293) deemed it fit to allow veterinary technicians to vaccinate their dogs. This is highly discouraged as only certified veterinarians should administer vaccines and biologics, and sign a vaccination certificate. Most dog owners were willing to take their dogs for free anti-rabies mass vaccinations in the state. While the level of education significantly affects the awareness rates of rabies (p < 0.001), it does not have an impact on the knowledge scores of the respondents (p = 0.829).

Although most of the respondents visited a health facility for the treatment of dog-bite wounds, others (20.9%, 1.8%, and 1.7%) still believed in traditional concoctions, spiritual interference, or did nothing to dog-bite incidents, respectively. This is a better attitude than reports of other studies in Nigeria (Niger and Bauchi states) where only 28.1% and 35.4% of the dog bite victims received PEP from a health facility respectively [12, 39]. This attitude was also consistent with the findings of Edukugbo et al. [25] and Christopher et al. [26]. Self-medication/treatment of bite wounds by some respondents may have been influenced by a low level of education, financial constraints, or ease and accessibility to a health facility. The World Health Organization (WHO) recommends wound washing and vaccination immediately after contact with a suspect rabid animal which can prevent almost 100% of rabies deaths [1, 35].

Few respondents owned at least one dog. Because many dogs were given out as gifts from friends and family, there is the potential of the spread of rabies from one environment to the other through this uncontrolled movement [40]. In these households, every member of the family takes care of the owned dog, and dogs were mostly fed family left-over meals. These results were consistent with reports from other parts of the country where all members of the family care for dogs [12, 39, 41]. Because such left-over meals were mostly insufficient, dogs were allowed to roam freely to supplement their feed. This increases the chances of provoked dog bite incidents and dog to dog transmission of rabies [12, 39–41].

The main strengths of this study were the wide geographical coverage and its timeliness in our effort to design a robust rabies control program in the state. However, the major limitation was that this study did not account for the inter-cluster correlation of respondents or clustering by locality.

## Conclusion

Although respondents showed low awareness and knowledge regarding canine rabies, the most obvious knowledge gaps were in the symptoms, preventive and control measures needed to eliminate canine rabies. Advocacy for an attitudinal change against all unorthodox treatment methods of dog-bites wounds should be enhanced. These unorthodox treatment practices result in gross under-reporting of cases, which undermines our concerted efforts of eliminating dog mediated human rabies in Kwara state.

## Supporting information

S1 File Survey material on “awareness and knowledge of canine rabies in Kwara state”. (DOCX)

S1 Fig Flow-diagram for the multi-stage sampling of respondents’ household used in this study. (DOCX)

S2 Fig Geo-coordinates of sampling points included in this study (n = 1,460). (DOCX)

S1 Table Practices associated with management of owned dogs in Kwara state. (DOCX)
