# Supplementary material for: Awareness and knowledge of canine rabies: A state-wide cross-sectional study in Nigeria
Source: PLoS One. 2021 Mar 3;16(3):e0247523. doi: 10.1371/journal.pone.0247523 (PMC7928438; doi:10.1371/journal.pone.0247523)
Supplement: S1 File — (DOCX) [file pone.0247523.s001.docx]

**Awareness and knowledge of canine rabies among residents of Kwara State**

This study aims to assess the awareness and knowledge of canine rabies among residents of Kwara State. The study is essential to formulate a robust state-wide rabies prevention and control strategy to achieve global zero human rabies death by 2030. This will take 3-5 minutes. Your participation is voluntary and you can withdraw at anytime. The information will be confidential and for research purposes only. For more information, please contact [ai.almustapha42@gmail.com](mailto:ai.almustapha42@gmail.com).

**CONSENT**

Do I have your permission to continue?

Yes (Append signature or thumbprint on consent sheet)

No

**SECTION A: RESPONDENTS BIO-DATA**

Age (years)? ………………………………………………

LGA……………………………………………………………

Gender:

Male

Female

What is your highest level of education?

Primary

Secondary

First degree

Master degree

PhD

If others, please specify

**SECTION B: Awareness of canine rabies**

Have you heard of rabies (locally called digbolugi, hawkan kare, gben bande)?

No (if no, skip to section D)

Yes (if yes, go to section C)

**SECTION C: Knowledge of canine rabies**

What is the cause of rabies?

Bacteria

Fungi

Protozoan

Virus

I don’t know

Which of these are the symptoms of Rabies?

Behavioural Changes

Dropped jaw

Fever

Hydrophobia

I don’t know

Inability to swallow

Paralysis

Pica

Seizures

Which of these is (are) the modes of transmission of rabies? (Select all that applies)

Blood

Contact

Dog bites

Penetration of open wound with dog saliva

I don’t know

What can be used to prevent transmission of rabies from dogs to human? (Select all that applies)

Antibiotics

Human vaccinations

Killing stray dogs

Mass dog vaccinations

I don’t know

Which of these is (are) can be used to control rabies in dogs? (Select all that applies)

Control Measures

Killing of stray-dogs

Mass dog vaccinations

Public awareness campaigns

Spaying

I don’t know

**SECTION D: Practices associated with management of owned dogs in Kwara state**

Do you have dogs in your premise?

Yes

No

What do you do when there is a dog-bite incident?

Take the victim to the spiritual healers

Take the victim to the hospital

I use traditional medicine

I practice self - treatment

Wash with soap and water

Nothing

Will you allow vet technicians to vaccinate your dog?

No

Yes

Do you confine your dog?

Always

I don’t know

Never

Partial

Who cares for the dog?

Children

Everybody

Father

Mother

What do you feed your dog?

Commercial dog feeds

Cook special pot

Family left-over

What is the source of your dog(s)?

Bought

I don’t know

Gift

Offspring of bitch

How is your dog depopulated?

I usually give away

I don't give out my dogs

I sell them

Do you think that the Veterinary services is adequate in your community?

No

Yes

Thank you for your time.
