# Supplementary figures and images for: Awareness and knowledge of canine rabies: A state-wide cross-sectional study in Nigeria
Source: PLoS One. 2021 Mar 3;16(3):e0247523. doi: 10.1371/journal.pone.0247523 (PMC7928438; doi:10.1371/journal.pone.0247523)

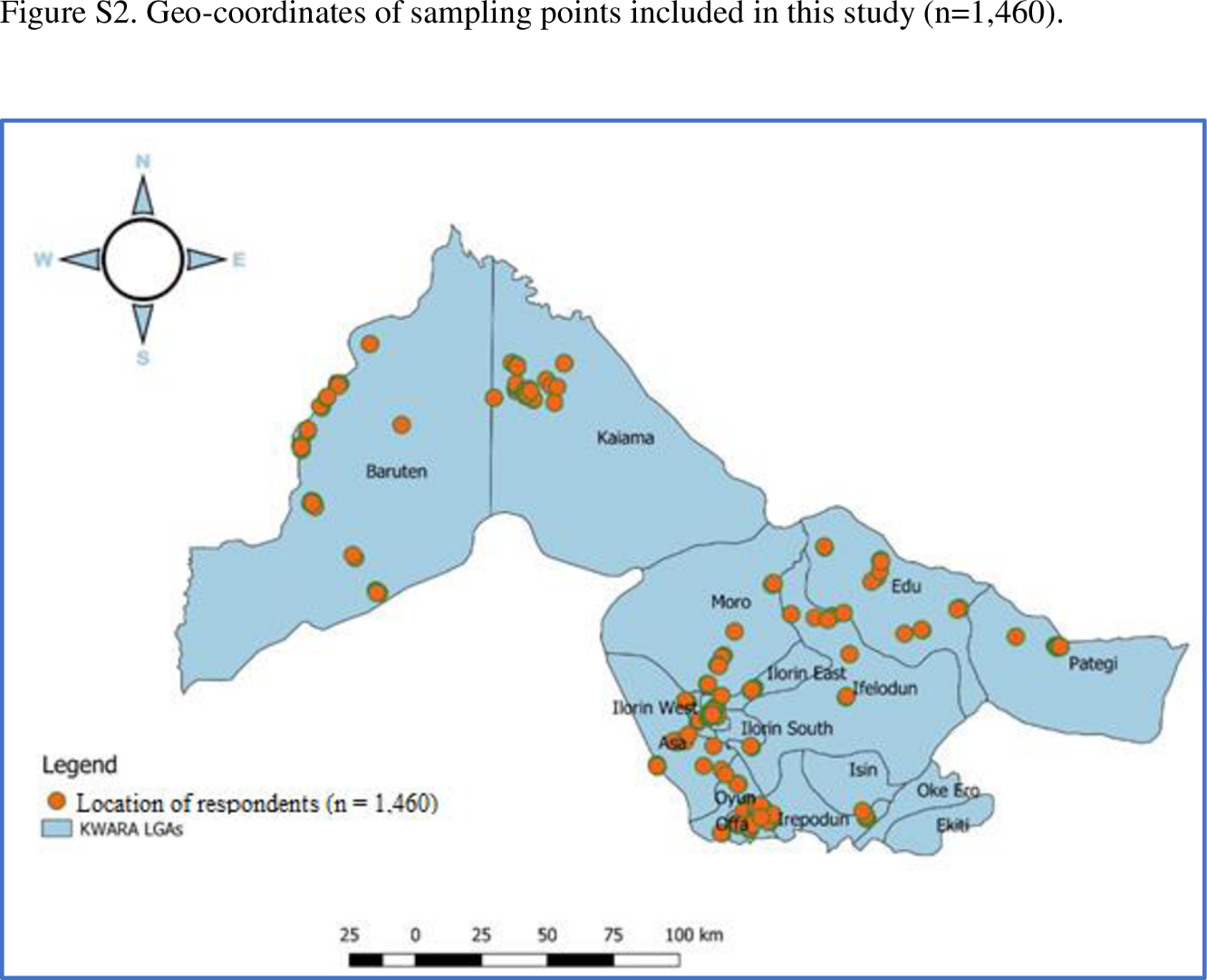

Supplement: S2 Fig — (DOCX) [file pone.0247523.s003.docx]
