# Supplementary material for: Awareness and knowledge of canine rabies: A state-wide cross-sectional study in Nigeria
Source: PLoS One. 2021 Mar 3;16(3):e0247523. doi: 10.1371/journal.pone.0247523 (PMC7928438; doi:10.1371/journal.pone.0247523)

SUPPLEMENTARY DATA

Figure S1. Flow-diagram for multi-stage sampling of respondents' household used in this study.

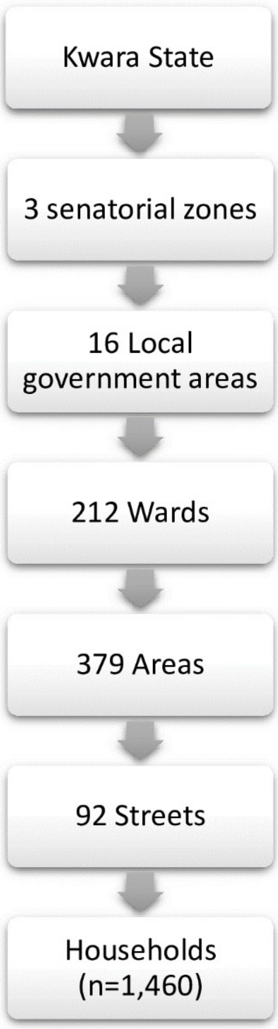

Supplement: Supplementary file 3 [file pone.0247523.s002.docx]
