# Supplementary material for: Awareness and knowledge of canine rabies: A state-wide cross-sectional study in Nigeria
Source: PLoS One. 2021 Mar 3;16(3):e0247523. doi: 10.1371/journal.pone.0247523 (PMC7928438; doi:10.1371/journal.pone.0247523)
Supplement: Supplementary file 4 [file pone.0247523.s004.docx]

Table S1. Practices associated with management of owned dogs in Kwara state.

Variable	Frequency (%)
What do you do when there is a dog-bite incident	
Take the victim to the spiritual healers	27 (1.8)
Take the victim to the hospital	960 (65.7)
I use traditional medicine	305 (20.9)
I practice self - treatment	446 (30.5)
Wash with soap and water	149 (10.2)
Nothing	25 (1.7)
Will you allow vet technicians to vaccinate your dog?	
No	65 (22.2)
Yes	228 (77.8)
Dogs in your household?	
Yes	293 (20.1)
No	1167 (79.9)
Confinement of dog	
Always	80 (27)
I don’t know	11 (4)
Never	152 (52)
Partial	50 (17)
Who cares for dog?	
Children	31 (11)
Everybody	200 (70)
Father	52 (18)
Mother	3 (1)
What do you feed your dog?	
Commercial dog feeds	20 (7)
Cook special pot	35 (12)
Family left-over	238 (81)
Source of dogs	
Bought	97 (32)
I don’t know	20 (7)
Gift	154 (51)
Offspring of bitch	31 (10)
How is your dog depopulated?	
Give away	36 (55)
I don't	10 (15)
Killed	4 (6)
Sold	15 (23)
Adequacy of Vet. Services in your community	
No	104 (36)
Yes	183 (64)
